# Fluorescence multispectral imaging-based diagnostic system for atherosclerosis

**DOI:** 10.1186/s12938-016-0220-z

**Published:** 2016-08-20

**Authors:** Cassandra Su Lyn Ho, Toshikatsu Horiuchi, Hiroaki Taniguchi, Araya Umetsu, Kohsuke Hagisawa, Keiichi Iwaya, Kanji Nakai, Amalina Azmi, Natasha Zulaziz, Azran Azhim, Nariyoshi Shinomiya, Yuji Morimoto

**Affiliations:** 1 Department of Electronic Systems Engineering, Malaysia-Japan International Institute of Technology, Universiti Teknologi Malaysia, 54100 Kuala Lumpur, Malaysia; 2 Department of Integrative Physiology and Bio-Nano Medicine, National Defense Medical College, Tokorozawa, Saitama 359-8513 Japan; 3 Department of Physiology, National Defense Medical College, Tokorozawa, Saitama 359-8513 Japan; 4 Department of Pathology, Sasaki Institute, Kyoundo Hospital, Chiyoda, Tokyo, 101-0062 Japan; 5 Department of Radiology, National Defense Medical College, Tokorozawa, Saitama 359-8513 Japan; 6 Department of Biotechnology, Kulliyyah of Science, International Islamic University Malaysia, 25200 Kuantan, Pahang Malaysia

**Keywords:** Atherosclerosis, Multispectral fluorescence imaging, Abdominal aorta, Coronary artery

## Abstract

**Background:**

Composition of atherosclerotic arterial walls is rich in lipids such as cholesterol, unlike normal arterial walls. In this study, we aimed to utilize this difference to diagnose atherosclerosis via multispectral fluorescence imaging, which allows for identification of fluorescence originating from the substance in the arterial wall.

**Methods:**

The inner surface of extracted arteries (rabbit abdominal aorta, human coronary artery) was illuminated by 405 nm excitation light and multispectral fluorescence images were obtained. Pathological examination of human coronary artery samples were carried out and thickness of arteries were calculated by measuring combined media and intima thickness.

**Results:**

The fluorescence spectra in atherosclerotic sites were different from those in normal sites. Multiple regions of interest (ROI) were selected within each sample and a ratio between two fluorescence intensity differences (where each intensity difference is calculated between an identifier wavelength and a base wavelength) from each ROI was determined, allowing for discrimination of atherosclerotic sites. Fluorescence intensity and thickness of artery were found to be significantly correlated.

**Conclusions:**

These results indicate that multispectral fluorescence imaging provides qualitative and quantitative evaluations of atherosclerosis and is therefore a viable method of diagnosing the disease.

**Electronic supplementary material:**

The online version of this article (doi:10.1186/s12938-016-0220-z) contains supplementary material, which is available to authorized users.

## Background

Cardiovascular diseases (CVD) are the leading cause of death globally and killed an approximate 17.5 million people worldwide in 2012; making up three in every 10 deaths [1]. Of this number, 7.4 million people died of ischemic heart disease. Atherosclerosis is a known precursor of ischemic heart disease and is defined as the thickening or stiffening of arteries with plaque—restricting blood flow from the heart to other organs and tissues. Over time, the progressive accumulation of plaque leads to rupture and thrombosis formation that can be life threatening. However, if detected early, atherosclerosis is reversible [2].

The use of intravascular imaging techniques currently utilized in diagnosing atherosclerosis can lead to procedure-related complications, long procedural times, and high expenditures [3]. In addition, existing imaging modalities that are frequently used in clinical practice, such as intravascular ultrasound and coronary angiography, do not present a comprehensive assessment of the arterial plaque and subsequently progression of disease as they are not able to distinguish among specific cellular and molecular biomarkers of atherosclerotic lesion. Hence, development of new and/or improved intravascular imaging modalities that are able to produce anatomical images along with compositional information are vital in advancing the diagnostic field. In the present study, we proposed diagnosis of atherosclerosis using multispectral imaging technology.

Arterial wall in atherosclerosis contains high levels of lipids, mostly cholesterol. As certain lipids contain intrinsic fluorescence properties, this suggests the potential of diagnosing atherosclerosis based on biochemical properties. Previous research has indicated that collagen, elastin, ceroid, and tryptophan (all present in human arterial wall) possess specific fluorescence spectral features [4]. Collagen and elastin are present as structural proteins in both normal and diseased arteries, where an increase in collagen has been noted in atherosclerotic arteries [5, 6]. Conversely, tryptophan and ceroid are only present in atherosclerotic lesion [7, 8]. These spectral changes in intrinsic tissue fluorescence can therefore be applied in diagnosis of atherosclerosis. Multispectral imaging is a technique of image processing and/or analysis often used in geographical analysis by satellites and also in remote sensing for detecting freshness of foods. This technology is also recently emerging as a diagnostic method in the field of medicine. The principle behind this method is such that when a sample is irradiated with excitation light, each point or pixel produces its corresponding reflection spectrum. In some cases, fluorescence spectrum is obtained. Therefore, spectral information can be obtained at each separate point (pixel) of the sample.

In this study, we hypothesized that multispectral fluorescence imaging can be used in detecting biochemical properties of arteries, which can subsequently be used in diagnosing atherosclerotic lesion.

## Methods

### Analysis of atherosclerotic aorta in rabbits

Four-week-old rabbits (*Oryctolagus cuniculus*) were administered with a 1 % cholesterol-containing feed for 27 weeks prior to aorta extraction. This resulted in atherosclerosis being developed in almost all the animals. For the purpose of this study, the abdominal aorta was extracted.

Isolated aorta was opened longitudinally along the ventral midline and gently rinsed blood with saline. The intimal side of the sample was irradiated with 405 nm excitation light. The multispectral fluorescence imaging system used had a spatial resolution of 1024 × 1024 pixels for a resulting fluorescence spectrum of approximately one million pixels, with each pixel having a fluorescence spectrum range of 450–800 nm with a resolution of 4 nm.

This study was conducted in accordance to the guidelines of the Institutional Review Board for the care of animals at the National Defense Medical College, Japan.

### Application to human atherosclerotic coronary artery

Coronary arteries were extracted from human cadavers and were opened longitudinally and gently rinsed blood with saline. Analyses were carried out on blood vessels in the luminal surface (*n* = 9).

The protocol was approved by the ethics committee at National Defense Medical College, Tokorozawa.

## Results

### Analysis of atherosclerosis using rabbit aorta

**Fig. 1:**
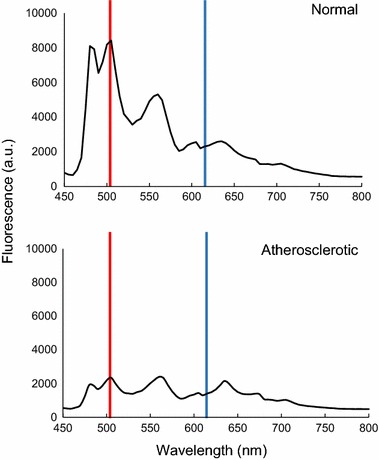
Fluorescence spectra of rabbit abdominal aorta samples. Resulting spectra of rabbit aorta samples indicated that fluorescence intensity at 505 nm (marked in *red line*) was four times higher in normal samples compared to atherosclerotic samples. Conversely, fluorescence intensity at 615 nm (marked in *blue line*) was the same in both normal and atherosclerotic samples

Using samples of rabbit abdominal aorta, comparison of the fluorescence spectrum was performed; the measurement of fluorescence intensity at 505 nm (FL_505_) revealed that the intensity of atherosclerotic sites decreased almost to a quarter than at normal sites (Fig. 1). This reduction in the fluorescence intensity correlated well with the presence of some chemical components such as excess collagen, tryptophan and ceroid, which quench the fluorescence radiation and are usually lacking in normal arterial walls [4–8]. Therefore, FL_505_ was considered to be a suitable indicator that identifies atherosclerotic changes in the walls of rabbit aorta. In contrast, fluorescence intensity at 615 nm (FL_615_) was found to be constant between normal and atherosclerotic sites. The lowest overall fluorescence value was also noted and marked as the “base” value. These three values at selected region of interests (ROIs) within each sample were recorded and used in the analysis of fluorescence spectra.

Thus introducing the analysis of fluorescence spectra the discrimination between normal and atherosclerotic sites in rabbit arteries was achieved. Atheromatous ratio (or AT ratio) is expressed by the following equation, Eq. 1:

1 $${\text{AT ratio}} = \frac{{FL_{505} - Base}}{{FL_{615} - Base}}$$

**Fig. 2:**
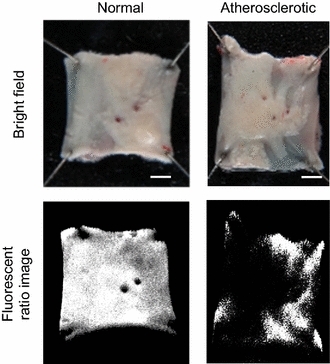
Reconstructed mapping of rabbit aorta samples. Difference in fluorescence intensity in normal and atherosclerotic artery samples allowed for discrimination between normal and diseased sites via ratio imaging. A pixel with the calculated AT ratio of ≥2 was depicted by *black* and a pixel with that of <2 was depicted by *white*. A pixel located in the area other than artery was depicted by black regardless of AT ratio. *Scale bar* 2 mm

The resulting AT ratio value was then used to produce reconstructed images, and the images were called “disease maps”. Reconstructed images by using AT ratio analysis clearly showed the difference between atherosclerotic and normal sites (Fig. 2).

### Analysis of atherosclerosis using human coronary artery

**Fig. 3:**
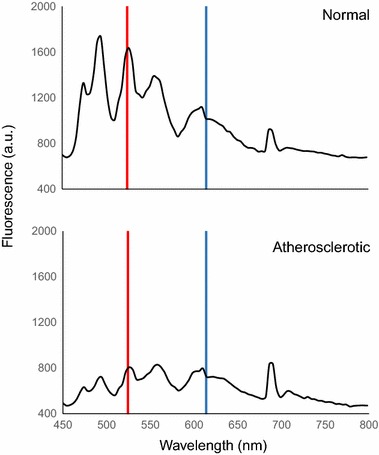
Fluorescence spectra of human coronary artery samples. Comparison of fluorescence spectra of human artery samples showed that fluorescence intensity at 526 nm (marked in *red line*) of atherosclerotic samples was a quarter than that of at normal samples. Fluorescence intensity at 618 nm (marked in *blue line*) was constant in both normal and atherosclerotic samples

Similar to the analysis of rabbit aorta, the samples of human coronary artery were processed to the spectral analysis. The fluorescence intensity at 526 nm (FL_526_) was markedly lower at atherosclerotic sites compared to normal sites and was thus used as an identifier (Fig. 3). On the other hand, the fluorescence intensity at 618 nm (FL_618_) was almost the same between atherosclerotic and normal coronary artery samples. In order to produce a corresponding “disease map” AT ratio values were then calculated as follows:

2 $${\text{AT ratio}} = \frac{{FL_{526} - Base}}{{FL_{618} - Base}}$$

**Fig. 4:**
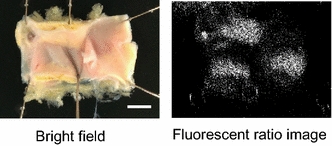
Reconstructed ratio image or “disease map” of a human coronary artery. Difference in fluorescence intensity in normal and atherosclerotic artery samples allowed for discrimination between normal and diseased sites via ratio imaging. A pixel with the calculated AT ratio of ≥2 was depicted by *black* and a pixel with that of <2 was depicted by *white*. A pixel located in the area other than artery was depicted by black regardless of AT ratio. *Scale bar* 4 mm

Similar to the rabbit samples, atherosclerotic sites were clearly distinguished in the reconstructed ratio image of human artery samples (Fig. 4).

### Correlation between AT ratio and arterial thickness

**Fig. 5:**
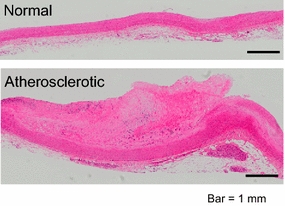
Human coronary artery samples stained with hematoxylin-eosin (HE). Atherosclerotic site shows a thickened wall due to plaque formation

**Fig. 6:**
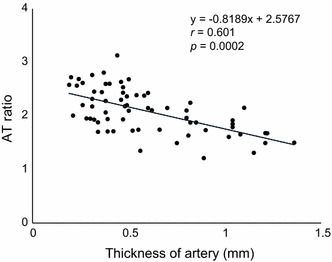
Scatter plot of AT ratio vs thickness of artery (mm). The data indicates a negative correlation between AT ratio and thickness of artery (*r* = 0.601, *p* = 0.0002). Thickness of artery can be estimated using the estimated regression model, *y* = −0.8189*x* + 2.5767

Pathological examination of human coronary artery samples with hematoxylin-eosin (HE) staining revealed that the thickness of arterial wall was highly associated with the depth of atheromatous plaque (Fig. 5). Glass slides were digitally scanned to provide a high resolution digital image using a virtual slide system (OlyVIA, Olympus), and the thickness of arterial walls was calculated by the sum of the measurements of both tunica media and intima. Results of the pathological examination and fluorescence spectrum analysis suggested an inverted linear correlation between the thickness of atheromatous lesion and the fluorescence intensity at the key indicator wavelength (FL_526_). Therefore, their correlation was confirmed by plotting AT ratio derived from the spectral data over the corresponding thickness of arterial wall at multiple points in each sample (*n* = 66) (Additional file 1). Statistical analysis revealed their linear correlation with a correlation coefficient of *r* = 0.60 and a significance of *p* = 0.00020 (Fig. 6).

**Fig. 7:**
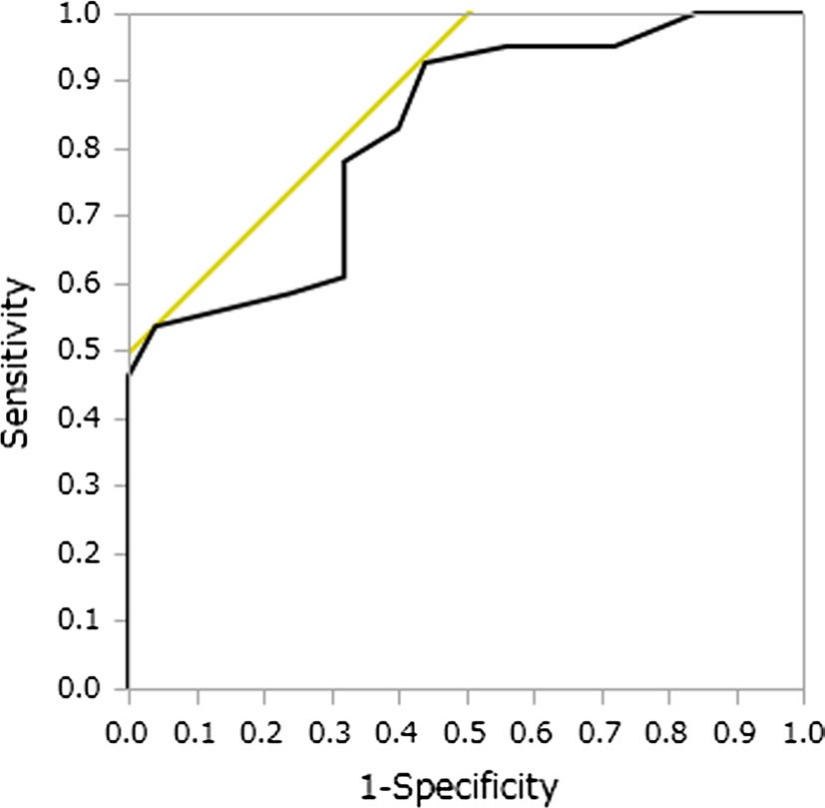
Receiver operating characteristic (ROC) curve indicating accuracy of arterial thickness for distinguishing normal and abnormal arterial wall. The resulting curve had an area under curve of 0.82, proving that measurement of arterial thickness is highly accurate in distinguishing between normal and abnormal samples. The *yellow line* in the plot is drawn at a 45° angle tangent to the ROC curve and indicates the probability threshold, which balances the frequency of false negatives and false positives

We further evaluated the diagnostic model by creating a receiver operating characteristic (ROC) curve using thickness of arterial wall and corresponding pathological judgment (normal or abnormal). As shown in Fig. 7, the resulting ROC curve indicated a highly accurate test with an area under curve of 0.82. Consequently, if an “abnormal” arterial wall judgment is set for AT ratio <2.1, our diagnostic tool will have a corresponding sensitivity of 0.78 and specificity of 0.68.

## Discussion

Current clinical methods for the detection of atherosclerotic lesions are limited and they often do not provide conclusive data, so the development of new technology is expected. The fluorescence imaging methodology used in this study may provide a quick and simple diagnostic way for both detection and quantification of atherosclerotic lesions. The application of multispectral fluorescent imaging to the analysis of atherosclerotic changes in arterial walls is based on the changes in biochemical composition of the luminal surface and vessel wall, which reflects the fluorescent status of the artery and provides information to interpret and validate the current status of the artery. This technology will be greatly helpful to assist the treatment of ischemic heart disease for atherosclerotic lesions.

We have demonstrated that multispectral fluorescence imaging is a useful and easy method for the detection of atherosclerotic sites. In the present study, the fluorescence peak at 505 nm in rabbit abdominal aorta and that at 526 nm in human coronary artery were key indicators to assess the extent of atherosclerosis. This indicator easily enables the discrimination of atherosclerotic tissues from normal arteries, and the depth and area of the atherosclerotic lesions can also be estimated quickly. As indicated in Table 1, candidate fluorophores that emit fluorescence at these wavelengths include collagen, elastin, FAD, etc. [9–17], all of which are important components of the normal arterial walls. The difference in peak wavelength between rabbit and human samples may be attributed to the difference in the ratio of collagen/elastin content in samples. Collagen content was probably higher in human coronary artery samples in this study since they were derived from aged patients while higher elastin content was anticipated in rabbit abdominal aorta because they were derived from 30-week-old rabbits (corresponding to 20–30 year old human). Additionally, the difference in peak wavelength may be related to the difference in pH values in the samples. Relatively small changes in pH are able to affect spectral characteristics of fluorescence, resulting in one fluorophore emitting fluorescence at different wavelengths [18].

**Table 1:** Candidate fluorophores with fluorescence emission ~505–526 nm

Fluorophores	Group	Excitation (nm)	Spectral characteristic (nm)	
			Emission range	Emission maxima
Ceroid	Ramanujam [9]	340–395	430–460, 540	540
	Richards-Kortum & Sevick-Muraca [10]	340–395	430–540, 540–640	640
Collagen	Richards-Kortum & Sevick-Muraca [10]	450	–	530
Elastin	Richards-Kortum & Sevick-Muraca [10]	410	–	500
	Kollias et al. [11]	420, 460	–	500, 540
FAD	Ramanujam [9]	450	–	535
	Richards-Kortum & Sevick-Muraca [10]	450	–	515
	Vo-Dinh [12]	440	–	520
	Zheng et al. [13]	410, 440	–	520
	Bachmann et al. [14]	450	–	535
NADH	Wu & Qu [15]	405	490–520	–
*β*-Carotene	Andersson-Engels et al. [16]	377	400–700	520
	Kandori et al. [17]	425	–	513

**Fig. 8:**
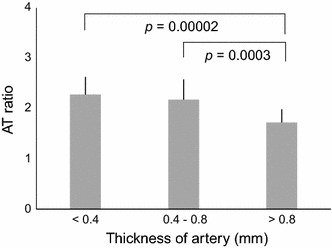
Correlation between AT ratio and thickness of atheromatous lesion. The estimated regression model can successfully estimate the relative thickness of artery based on AT ratio

Furthermore, this study using multispectral fluorescence imaging suggests the possibility of assessing of the qualitative evaluation as well as quantitative evaluation of atherosclerosis. In a study conducted to investigate the association between stroke and arterial thickness, it was reported that at arterial (intima-media) thickness of 0.53 ± 0.03 and 0.71 ± 0.02 mm, risk of ischemic stroke doubled and quadrupled respectively (based on control of 0.39 ± 0.07 mm) [19]. Correspondingly, the atherosclerosis risk in communities (ARIC) Study indicated that the relative risk of ischemic stroke is the lowest and highest at arterial thickness of <0.59 and ≥0.70 mm respectively [20]. Several other studies have also shown similar results [21, 22]. Based on the fluorescence data from the samples, we were able to calculate AT ratio (that was plotted against corresponding measured thickness of arteries) and produced a regression model for its estimation, which successfully showed positive correlation between the estimation of the relative thickness of the artery and the progression status of the atherosclerotic lesion (Fig. 8). The ability of this system to effectively measure thickness of artery and subsequently progression of the disease will be valuable when translated to an endoscope-based system for in vivo diagnosis.

## Conclusions

We have demonstrated that multispectral fluorescence imaging is a new method of detecting atherosclerosis sites; it is useful for both quantitative and qualitative evaluation of the disease. With further improvements, we believe that the application of this technology to an endoscope-based diagnostic system will be beneficial in constructing a new intravascular diagnostic tool for the in vivo detection of atherosclerotic lesions.

## Additional file

10.1186/s12938-016-0220-z Raw and analyzed data.

## Abbreviations

ROI: region of interest
CVD: cardiovascular diseases
FL: fluorescence intensity
AT: atheromatous
HE: hematoxylin-eosin
ROC: receiver operating characteristic
FAD: flavin adenine dinucleotide
ARIC: atherosclerosis risk in communities
